# Targeting transthyretin (TTR) stability for amyloid cardiomyopathy: a landmark shift in heart failure therapy

**DOI:** 10.1097/MS9.0000000000003762

**Published:** 2025-08-29

**Authors:** Muddassir Khalid, Taha Kassim Dohadwala, Ghufran Saeed Rizvi, Supoma Gosh Ria

**Affiliations:** a Department of Medicine, Nishtar Medical University, Multan, Pakistan; b Department of Medicine, David Tvildiani Medical University, Tbilisi, Georgia; c Department of Medicine, Shifa College of Medicine, Islamabad, Pakistan; d Mugda Medical College, University of Dhaka, Bangladesh

Transthyretin amyloid cardiomyopathy (ATTR-CM) is now being increasingly recognized as a previously underdiagnosed cause of heart failure in later life, occurring in two forms – hereditary (ATTRv-CM) and wild type (ATTRwt-CM), both with poor prognoses and a median survival rate of 2–6 years. ATTR-CM is the consequence of extracellular deposition of misfolded transthyretin (TTR) protein in the myocardium, progressively leading to restrictive cardiomyopathy, diastolic dysfunction, and heart failure. However, the introduction of a TTR stabilizer, based on the ATTR-ACT trial, is expected to be a turning point in disease-specific treatment for ATTR-CM. The recently conducted ATTR-ACT trial has proven that TTR stabilizers reduce mortality by 30% and cardiovascular-related hospitalizations by 32% over placebo. This means that patients treated with TTR stabilizers had a significantly lower risk of death and fewer hospital visits due to heart-related issues, with excellent treatment adherence (97.2% for TTR stabilizer). These outcomes were particularly evident in NYHA class I–II symptom patients, though further investigations continue to define its role in class III heart failure^[1]^. Tafamidis is one of the substantial drugs among TTR kinetic stabilizers that bind to the thyroxine-binding sites on TTR and inhibit tetramer dissociation, the rate-limiting step in amyloid fibril formation^[2,3]^.

Shah *et al* further reported tafamidis’s (80 mg) role in stabilizing left ventricular ejection fraction (LVEF), with marked echocardiographic improvements in left ventricular (LV) stroke volume, global longitudinal strain, and *E*/*e*′ ratios^[4]^. Drachma *et al* substantiated long-term efficacy, finding enhanced survival with a 47% decrease in the risk of mortality and improved quality of life after 60 months (approximately 5 years) in patients with baseline LVEF <50% and ≥50%^[5]^. Despite promising trial studies, several measures should be taken to overcome diagnostic delays and ensure the timely administration of TTR stabilizers. There is a need for clinical education on red-flag signs, such as unexplained LV hypertrophy, bilateral carpal tunnel syndrome, or atrial arrhythmias in elderly patients^[3]^. Screening should be given priority in patients with heart failure symptoms, particularly decreased or preserved LVEF^[4]^. Encouraging advanced imaging, such as bone scintigraphy, which can detect amyloid deposits in the heart, and genetic testing, which can identify the specific mutation causing ATTR-CM, can facilitate early detection of the disease and enable the initiation of TTR stabilizers before irreversible cardiac damage^[1,2]^. We seek to highlight the therapeutic potential of TTR stabilizers as an effective treatment option for AATR-CM by reflecting its role in improving survival, cardiovascular-related outcomes, and quality of life in the eligible population.

However, the affordability and availability of such treatment options are still significant problems, particularly in low- and middle-income countries. Nevertheless, future research to evaluate safety and effectiveness and comparative trials with other new therapies, such as gene silencers and CRISPR-based therapies, may establish the accurate position of TTR stabilizers in the dynamic treatment strategy.

TITAN Guidelines: This manuscript complies with TITAN Guidelines, 2025, declaring no use of AI^[6]^.

## Data Availability

Not applicable.
